# COVID-19 due to the B.1.617.2 (Delta) variant compared to B.1.1.7 (Alpha) variant of SARS-CoV-2: a prospective observational cohort study

**DOI:** 10.1038/s41598-022-14016-0

**Published:** 2022-06-28

**Authors:** Kerstin Kläser, Erika Molteni, Mark Graham, Liane S. Canas, Marc F. Österdahl, Michela Antonelli, Liyuan Chen, Jie Deng, Benjamin Murray, Eric Kerfoot, Jonathan Wolf, Anna May, Ben Fox, Joan Capdevila, The COVID-19 Genomics U. K. (COG-UK) Consortium, David M. Aanensen, Khalil Abudahab, Helen Adams, Alexander Adams, Safiah Afifi, Dinesh Aggarwal, Shazaad S. Y. Ahmad, Louise Aigrain, Adela Alcolea-Medina, Nabil-Fareed Alikhan, Elias Allara, Roberto Amato, Adrienn Angyal, Tara Annett, Stephen Aplin, Cristina V. Ariani, Hibo Asad, Amy Ash, Paula Ashfield, Fiona Ashford, Laura Atkinson, Stephen W. Attwood, Cressida Auckland, Alp Aydin, David J. Baker, Paul Baker, Carlos E. Balcazar, Jonathan Ball, Jeffrey C. Barrett, Magdalena Barrow, Edward Barton, Matthew Bashton, Andrew R. Bassett, Rahul Batra, Chris Baxter, Nadua Bayzid, Charlotte Beaver, Angela H. Beckett, Shaun M. Beckwith, Luke Bedford, Robert Beer, Andrew Beggs, Katherine L. Bellis, Louise Berry, Beatrice Bertolusso, Angus Best, Emma Betteridge, David Bibby, Kelly Bicknell, Debbie Binns, Alec Birchley, Paul W. Bird, Chloe Bishop, Rachel Blacow, Victoria Blakey, Beth Blane, Frances Bolt, James Bonfield, Stephen Bonner, David Bonsall, Tim Boswell, Andrew Bosworth, Yann Bourgeois, Olivia Boyd, Declan T. Bradley, Cassie Breen, Catherine Bresner, Judith Breuer, Stephen Bridgett, Iraad F. Bronner, Ellena Brooks, Alice Broos, Julianne R. Brown, Giselda Bucca, Sarah L. Buchan, David Buck, Matthew Bull, Phillipa J. Burns, Shirelle Burton-Fanning, Timothy Byaruhanga, Matthew Byott, Sharon Campbell, Alessandro M. Carabelli, James S. Cargill, Matthew Carlile, Sílvia F. Carvalho, Anna Casey, Anibolina Castigador, Jana Catalan, Vicki Chalker, Nicola J. Chaloner, Meera Chand, Joseph G. Chappell, Themoula Charalampous, Wendy Chatterton, Yasmin Chaudhry, Carol M. Churcher, Gemma Clark, Phillip Clarke, Benjamin J. Cogger, Kevin Cole, Jennifer Collins, Rachel Colquhoun, Thomas R. Connor, Kate F. Cook, Jason Coombes, Sally Corden, Claire Cormie, Nicholas Cortes, Marius Cotic, Seb Cotton, Simon Cottrell, Lindsay Coupland, MacGregor Cox, Alison Cox, Noel Craine, Liam Crawford, Aidan Cross, Matthew R. Crown, Dorian Crudgington, Nicola Cumley, Tanya Curran, Martin D. Curran, Ana da Silva Filipe, Gavin Dabrera, Alistair C. Darby, Rose K. Davidson, Alisha Davies, Robert M. Davies, Thomas Davis, Daniela de Angelis, Elen De Lacy, Leonardo de Oliveira Martins, Thushan I. de Silva, Johnny Debebe, Rebecca Denton-Smith, Samir Dervisevic, Rebecca Dewar, Jayasree Dey, Joana Dias, Donald Dobie, Matthew J. Dorman, Fatima Downing, Megan Driscoll, Louis du Plessis, Nichola Duckworth, Jillian Durham, Kirstine Eastick, Lisa J. Easton, Richard Eccles, Jonathan Edgeworth, Sue Edwards, Kate El Bouzidi, Sahar Eldirdiri, Nicholas Ellaby, Scott Elliott, Gary Eltringham, Leah Ensell, Michelle J. Erkiert, Marina Escalera Zamudio, Sarah Essex, Johnathan M. Evans, Cariad Evans, William Everson, Derek J. Fairley, Karlie Fallon, Arezou Fanaie, Ben W. Farr, Christopher Fearn, Theresa Feltwell, Lynne Ferguson, Laia Fina, Flavia Flaviani, Vicki M. Fleming, Sally Forrest, Ebenezer Foster-Nyarko, Benjamin H. Foulkes, Luke Foulser, Mireille Fragakis, Dan Frampton, Sarah Francois, Christophe Fraser, Timothy M. Freeman, Helen Fryer, Marc Fuchs, William Fuller, Kavitha Gajee, Katerina Galai, Abbie Gallagher, Eileen Gallagher, Michael D. Gallagher, Marta Gallis, Amy Gaskin, Bree Gatica-Wilcox, Lily Geidelberg, Matthew Gemmell, Iliana Georgana, Ryan P. George, Laura Gifford, Lauren Gilbert, Sophia T. Girgis, Sharon Glaysher, Emily J. Goldstein, Tanya Golubchik, Andrea N. Gomes, Sónia Gonçalves, Ian G. Goodfellow, Scott Goodwin, Salman Goudarzi, Marina Gourtovaia, Clive Graham, Lee Graham, Paul R. Grant, Luke R. Green, Angie Green, Jane Greenaway, Richard Gregory, Martyn Guest, Rory N. Gunson, Ravi K. Gupta, Bernardo Gutierrez, Sam T. Haldenby, William L. Hamilton, Samantha E. Hansford, Tanzina Haque, Kathryn A. Harris, Ian Harrison, Ewan M. Harrison, Jennifer Hart, John A. Hartley, William T. Harvey, Matthew Harvey, Mohammed O. Hassan-Ibrahim, Judith Heaney, Thomas Helmer, John H. Henderson, Andrew R. Hesketh, Jessica Hey, David Heyburn, Ellen E. Higginson, Verity Hill, Jack D. Hill, Rachel A. Hilson, Ember Hilvers, Matthew T. G. Holden, Amy Hollis, Christopher W. Holmes, Nadine Holmes, Alison H. Holmes, Richard Hopes, Hailey R. Hornsby, Myra Hosmillo, Catherine Houlihan, Hannah C. Howson-Wells, Sharon N. Hsu, Jonathan Hubb, Hannah Huckson, Warwick Hughes, Joseph Hughes, Margaret Hughes, Stephanie Hutchings, Giles Idle, Chris J. Illingworth, Robert Impey, Dianne Irish-Tavares, Miren Iturriza-Gomara, Rhys Izuagbe, Chris Jackson, Ben Jackson, Leigh M. Jackson, Kathryn A. Jackson, David K. Jackson, Aminu S. Jahun, Victoria James, Keith James, Christopher Jeanes, Aaron R. Jeffries, Sarah Jeremiah, Andrew Jermy, Michaela John, Rob Johnson, Kate Johnson, Ian Johnston, Owen Jones, Sophie Jones, Hannah Jones, Christopher R. Jones, Neil Jones, Amelia Joseph, Sarah Judges, Gemma L. Kay, Sally Kay, Jon-Paul Keatley, Alexander J. Keeley, Anita Kenyon, Leanne M. Kermack, Manjinder Khakh, Stephen P. Kidd, Maimuna Kimuli, Stuart Kirk, Christine Kitchen, Katie Kitchman, Bridget A. Knight, Cherian Koshy, Moritz U. G. Kraemer, Sara Kumziene-Summerhayes, Dominic Kwiatkowski, Angie Lackenby, Kenneth G. Laing, Temi Lampejo, Cordelia F. Langford, Deborah Lavin, Andrew I. Lawton, Jack C. D. Lee, David Lee, Stefanie V. Lensing, Steven Leonard, Lisa J. Levett, Thanh Le-Viet, Jonathan Lewis, Kevin Lewis, Jennifier Liddle, Steven Liggett, Patrick J. Lillie, Benjamin B. Lindsey, Michelle M. Lister, Rich Livett, Stephanie Lo, Nicholas J. Loman, Matthew W. Loose, Stavroula F. Louka, Katie F. Loveson, Sarah Lowdon, Hannah Lowe, Helen L. Lowe, Anita O. Lucaci, Catherine Ludden, Jessica Lynch, Ronan A. Lyons, Katrina Lythgoe, Nicholas W. Machin, George MacIntyre-Cockett, Andrew Mack, Ben Macklin, Alasdair Maclean, Emily Macnaughton, Pinglawathee Madona, Mailis Maes, Laurentiu Maftei, Adhyana I. K. Mahanama, Tabitha W. Mahungu, Daniel Mair, Joshua Maksimovic, Cassandra S. Malone, Daniel Maloney, Nikos Manesis, Robin Manley, Anna Mantzouratou, Angela Marchbank, Arun Mariappan, Inigo Martincorena, Rocio T. Martinez Nunez, Alison E. Mather, Patrick Maxwell, Megan Mayhew, Tamyo Mbisa, Clare M. McCann, Shane A. McCarthy, Kathryn McCluggage, Patrick C. McClure, J. T. McCrone, Martin P. McHugh, James P. McKenna, Caoimhe McKerr, Georgina M. McManus, Claire L. McMurray, Claire McMurray, Alan McNally, Lizzie Meadows, Nathan Medd, Oliver Megram, Mirko Menegazzo, Ian Merrick, Stephen L. Michell, Michelle L. Michelsen, Mariyam Mirfenderesky, Jeremy Mirza, Julia Miskelly, Emma Moles-Garcia, Robin J. Moll, Zoltan Molnar, Irene M. Monahan, Matteo Mondani, Siddharth Mookerjee, Christopher Moore, Jonathan Moore, Nathan Moore, Catherine Moore, Helen Morcrette, Sian Morgan, Mari Morgan, Matilde Mori, Arthur Morriss, Samuel Moses, Craig Mower, Peter Muir, Afrida Mukaddas, Florence Munemo, Robert Munn, Abigail Murray, Leanne J. Murray, Darren R. Murray, Manasa Mutingwende, Richard Myers, Eleni Nastouli, Gaia Nebbia, Andrew Nelson, Charlotte Nelson, Sam Nicholls, Jenna Nichols, Roberto Nicodemi, Kyriaki Nomikou, Justin O’Grady, Sarah O’Brien, Mina Odedra, Natasha Ohemeng-Kumi, Karen Oliver, Richard J. Orton, Husam Osman, Áine O’Toole, Nicole Pacchiarini, Debra Padgett, Andrew J. Page, Emily J. Park, Naomi R. Park, Matthew D. Parker, Surendra Parmar, David G. Partridge, David Pascall, Amita Patel, Bindi Patel, Steve Paterson, Brendan A. I. Payne, Sharon J. Peacock, Clare Pearson, Emanuela Pelosi, Benita Percival, Jon Perkins, Malorie Perry, Malte L. Pinckert, Steven Platt, Olga Podplomyk, Manoj Pohare, Marcus Pond, Cassie F. Pope, Radoslaw Poplawski, Jessica Powell, Jennifer Poyner, Liam Prestwood, Anna Price, James R. Price, Jacqui A. Prieto, David T. Pritchard, Sophie J. Prosolek, Georgia Pugh, Monika Pusok, Oliver G. Pybus, Hannah M. Pymont, Michael A. Quail, Joshua Quick, Clara Radulescu, Jayna Raghwani, Manon Ragonnet-Cronin, Lucille Rainbow, Diana Rajan, Shavanthi Rajatileka, Newara A. Ramadan, Andrew Rambaut, John Ramble, Paul A. Randell, Paul Randell, Liz Ratcliffe, Veena Raviprakash, Mohammad Raza, Nicholas M. Redshaw, Sara Rey, Nicola Reynolds, Alex Richter, David L. Robertson, Esther Robinson, Samuel C. Robson, Fiona Rogan, Stefan Rooke, Will Rowe, Sunando Roy, Steven Rudder, Chris Ruis, Steven Rushton, Felicity Ryan, Kordo Saeed, Buddhini Samaraweera, Christine M. Sambles, Roy Sanderson, Theo Sanderson, Fei Sang, Thea Sass, Emily Scher, Garren Scott, Carol Scott, Jasveen Sehmi, Sharif Shaaban, Divya Shah, Jessica Shaw, Ekaterina Shelest, James G. Shepherd, Liz A. Sheridan, Nicola Sheriff, Lesley Shirley, John Sillitoe, Siona Silviera, David A. Simpson, Aditi Singh, Dawn Singleton, Timofey Skvortsov, Tim J. Sloan, Graciela Sluga, Ken Smith, Kim S. Smith, Perminder Smith, Darren L. Smith, Louise Smith, Colin P. Smith, Nikki Smith, Katherine L. Smollett, Luke B. Snell, Thomas Somassa, Joel Southgate, Karla Spellman, Michael H. Spencer Chapman, Lewis G. Spurgin, Moira J. Spyer, Rachael Stanley, William Stanley, Thomas D. Stanton, Igor Starinskij, Joanne Stockton, Susanne Stonehouse, Nathaniel Storey, David J. Studholme, Malur Sudhanva, Emma Swindells, Yusri Taha, Ngee Keong Tan, Julian W. Tang, Miao Tang, Ben E. W. Taylor, Joshua F. Taylor, Sarah Taylor, Ben Temperton, Kate E. Templeton, Claire Thomas, Laura Thomson, Emma C. Thomson, Alicia Thornton, Scott A. J. Thurston, John A. Todd, Rachael Tomb, Lily Tong, Gerry Tonkin-Hill, M. Estee Torok, Jaime M. Tovar-Corona, Amy Trebes, Alexander J. Trotter, Ioulia Tsatsani, Robyn Turnbull, Lance Turtle, Katherine A. Twohig, Helen Umpleby, Anthony P. Underwood, Edith E. Vamos, Tetyana I. Vasylyeva, Sreenu Vattipally, Gabrielle Vernet, Barry B. Vipond, Erik M. Volz, Sarah Walsh, Dennis Wang, Ben Warne, Joanna Warwick-Dugdale, Elizabeth Wastnedge, Joanne Watkins, Louisa K. Watson, Sheila Waugh, Hermione J. Webster, Danni Weldon, Elaine Westwick, Thomas Whalley, Helen Wheeler, Mark Whitehead, Max Whiteley, Andrew Whitwham, Claudia Wierzbicki, Nicholas J. Willford, Lesley-Anne Williams, Rebecca Williams, Cheryl Williams, Chris Williams, Charlotte A. Williams, Rachel J. Williams, Thomas Williams, Catryn Williams, Kathleen A. Williamson, Eleri Wilson-Davies, Eric Witele, Karen T. Withell, Adam A. Witney, Paige Wolverson, Nick Wong, Trudy Workman, Victoria Wright, Derek W. Wright, Tim Wyatt, Sarah Wyllie, Li Xu-McCrae, Mehmet Yavus, Geraldine Yaze, Corin A. Yeats, Gonzalo Yebra, Wen C. Yew, Gregory R. Young, Jamie Young, Alex E. Zarebski, Peijun Zhang, Marc Modat, Alexander Hammers, Tim D. Spector, Claire J. Steves, Carole H. Sudre, Sebastien Ourselin, Emma L. Duncan

**Affiliations:** 1 School of Biomedical Engineering and Imaging Sciences, King’s College London, London, UK; 2 Department of Twin Research and Genetic Epidemiology, School of Life Course Sciences, Faculty of Life Sciences and Medicine, King’s College London, St Thomas’ Hospital Campus, 3rd Floor South Wing Block D, Westminster Bridge Road, London, SE1 7EH UK; 3 Department of Aging and Health, Guy’s and St Thomas’ NHS Foundation Trust, London, UK; 4 ZOE Limited, London, UK; 5 King’s College London & Guy’s and St Thomas’ PET Centre, London, UK; 6 MRC Unit for Lifelong Health and Ageing, Department of Population Health Sciences, University College London, London, UK; 7 Centre for Medical Image Computing, Department of Computer Science, University College London, London, UK; 8 Department of Endocrinology, Guy’s and St Thomas’ NHS Foundation Trust, London, UK; 9 Centre for Genomic Pathogen Surveillance, University of Oxford, Oxford, UK; 10 Betsi Cadwaladr University Health Board, Bangor, UK; 11 Public Health Wales, Cardiff, UK; 12 Cardiff and Vale University Health Board, Cardiff, UK; 13 Department of Medicine, University of Cambridge, Cambridge, UK; 14 Manchester University NHS Foundation Trust, Manchester, UK; 15 Wellcome Sanger Institute, Manchester, UK; 16 Guy’s and St. Thomas’ NHS Foundation Trust, London, UK; 17 Quadram Institute Bioscience, Norwich, UK; 18 Public Health England, London, UK; 19 University of Sheffield, Sheffield, UK; 20 University Hospital Southampton NHS Foundation Trust, Southampton, UK; 21 Barking, Havering and Redbridge University Hospitals NHS Trust, Romford, UK; 22 The Royal Wolverhampton NHS Trust, Wolverhampton, UK; 23 Turnkey Laboratory, University of Birmingham, Birmingham, UK; 24 Great Ormond Street Hospital for Children NHS Foundation Trust, London, UK; 25 Department of Zoology, University of Oxford, Oxford, UK; 26 Royal Devon and Exeter NHS Foundation Trust, Wonford, UK; 27 South Tees Hospitals NHS Foundation Trust, Middlesbrough, UK; 28 University of Edinburgh, Edinburgh, UK; 29 Virology, School of Life Sciences, Queens Medical Centre, University of Nottingham, Nottingham, UK; 30 Bournemouth University, Poole, UK; 31 North Cumbria Integrated Care NHS Foundation Trust, Cumbria, UK; 32 Hub for Biotechnology in the Built Environment, Northumbria University, Newcastle upon Tyne, UK; 33 Centre for Clinical Infection and Diagnostics Research, Department of Infectious Diseases, Guy’s and St Thomas’ NHS Foundation Trust, London, UK; 34 Queen’s University Belfast, Belfast, UK; 35 University College London Hospitals NHS Foundation Trust, London, UK; 36 Centre for Enzyme Innovation, University of Portsmouth, Portsmouth, UK; 37 Blackpool Teaching Hospitals NHS Foundation Trust, Blackpool, UK; 38 East Suffolk and North Essex NHS Foundation Trust, Colchester, UK; 39 University of Birmingham, Birmingham, UK; 40 Clinical Microbiology Department, Queens Medical Centre, Nottingham University Hospitals NHS Trust, Nottingham, UK; 41 Hampshire Hospitals NHS Foundation Trust, Basingstoke, UK; 42 Public Health England, Colindale, UK; 43 Portsmouth Hospitals University NHS Trust, Portsmouth, UK; 44 Microbiology Department, Buckinghamshire Healthcare NHS Trust, Amersham, UK; 45 Clinical Microbiology, University Hospitals of Leicester NHS Trust, Leicester, UK; 46 NHS Greater Glasgow and Clyde, Glasgow, UK; 47 County Durham and Darlington NHS Foundation Trust, Darlington, UK; 48 Imperial College Healthcare NHS Trust, London, UK; 49 Big Data Institute, Nuffield Department of Medicine, University of Oxford, Oxford, UK; 50 School of Biological Sciences, University of Portsmouth, Portsmouth, UK; 51 Imperial College London, London, UK; 52 Public Health Agency, Northern Ireland, Belfast, UK; 53 Liverpool Clinical Laboratories, Liverpool, UK; 54 Cardiff University, Cardiff, UK; 55 MRC-University of Glasgow Centre for Virus Research, Glasgow, UK; 56 University of Brighton, Brighton, UK; 57 Wellcome Centre for Human Genetics, Nuffield Department of Medicine, University of Oxford, Oxford, UK; 58 Hull University Teaching Hospitals NHS Trust, Hull, UK; 59 The Newcastle Upon Tyne Hospitals NHS Foundation Trust, Newcastle upon Tyne, UK; 60 University College London Hospital Advanced Pathogen Diagnostics Unit, London, UK; 61 East Sussex Healthcare NHS Trust, Seaford, UK; 62 Deep Seq, School of Life Sciences, Queens Medical Centre, University of Nottingham, Nottingham, UK; 63 Queen Elizabeth Hospital, Birmingham, Birmingham, UK; 64 Isle of Wight NHS Trust, Newport, UK; 65 Whittington Health NHS Trust, London, UK; 66 University Hospitals Sussex NHS Foundation Trust, Southampton, UK; 67 Health Services Laboratories, London, UK; 68 Division of Virology, Department of Pathology, University of Cambridge, Cambridge, UK; 69 Path Links, Northern Lincolnshire and Goole NHS Foundation Trust, London, UK; 70 Northumbria Healthcare NHS Foundation Trust, Newcastle upon Tyne, UK; 71 School of Pharmacy & Biomedical Sciences, University of Portsmouth, Portsmouth, UK; 72 University of Cambridge, Cambridge, UK; 73 NHS Lothian, Edinburgh, UK; 74 Norfolk and Norwich University Hospitals NHS Foundation Trust, Norwich, UK; 75 North Middlesex University Hospital NHS Trust, London, UK; 76 Northumbria University, Newcastle upon Tyne, UK; 77 University Hospitals Dorset NHS Foundation Trust, London, UK; 78 Belfast Health & Social Care Trust, Belfast, UK; 79 Public Health England, Cambridge, Cambridge, UK; 80 University of Liverpool, Liverpool, UK; 81 University of East Anglia, Norwich, UK; 82 Department of Microbiology, Kettering General Hospital, Kettering, UK; 83 MRC Biostatistics Unit, University of Cambridge, Cambridge, UK; 84 North Tees and Hartlepool NHS Foundation Trust, Durham, UK; 85 King’s College Hospital NHS Foundation Trust, London, UK; 86 Sheffield Teaching Hospitals NHS Foundation Trust, Sheffield, UK; 87 Watford General Hospital, Watford, UK; 88 West of Scotland Specialist Virology Centre, NHS Greater Glasgow and Clyde, Glasgow, UK; 89 Guy’s and St. Thomas’ Biomedical Research Centre, London, UK; 90 Division of Infection and Immunity, University College London, London, UK; 91 The Royal Marsden NHS Foundation Trust, London, UK; 92 Department of Infectious Diseases and Microbiology, Cambridge University Hospitals NHS Foundation Trust, Cambridge, UK; 93 Royal Free London NHS Foundation Trust, London, UK; 94 Public Health Scotland, London, UK; 95 University of Exeter, Exeter, UK; 96 The Queen Elizabeth Hospital King’s Lynn NHS Foundation Trust, King’s Lynn, UK; 97 Institute for Infection and Immunity, St George’s University of London, London, UK; 98 Southwest Pathology Services, Taunton, UK; 99 Institute of Microbiology and Infection, University of Birmingham, Birmingham, UK; 100 East Kent Hospitals University NHS Foundation Trust, London, UK; 101 Swansea University, Swansea, UK; 102 King’s College London, London, UK; 103 Gateshead Health NHS Foundation Trust, Gateshead, UK; 104 University of Southampton, Southampton, UK; 105 Newcastle University, Newcastle upon Tyne, UK; 106 Infection Care Group, St George’s University Hospitals NHS Foundation Trust, London, UK; 107 Royal Brompton and Harefield Hospitals, Harefield, UK; 108 Cambridge Stem Cell Institute, University of Cambridge, Cambridge, UK; 109 Maidstone and Tunbridge Wells NHS Trust, Royal Tunbridge Wells, UK; 110 Norfolk County Council, Norfolk, UK; 111 Institute of Biodiversity, Animal Health & Comparative Medicine, Glasgow, UK; 112 Great Ormond Street Institute of Child Health (GOS ICH), University College London (UCL), London, UK; 113 Department of Microbiology, South West London Pathology, London, UK; 114 Cambridge University Hospitals NHS Foundation Trust, Cambridge, UK; 115 Microbiology, Royal Oldham Hospital, Oldham, UK; 116 Heartlands Hospital, Birmingham, Birmingham, UK

**Keywords:** Comorbidities, Fatigue, Fever, Nausea, Pain, Pruritus, Respiratory signs and symptoms, Skin manifestations

## Abstract

The Delta (B.1.617.2) variant was the predominant UK circulating SARS-CoV-2 strain between May and December 2021. How Delta infection compares with previous variants is unknown. This prospective observational cohort study assessed symptomatic adults participating in the app-based COVID Symptom Study who tested positive for SARS-CoV-2 from May 26 to July 1, 2021 (Delta overwhelmingly the predominant circulating UK variant), compared (1:1, age- and sex-matched) with individuals presenting from December 28, 2020 to May 6, 2021 (Alpha (B.1.1.7) the predominant variant). We assessed illness (symptoms, duration, presentation to hospital) during Alpha- and Delta-predominant timeframes; and transmission, reinfection, and vaccine effectiveness during the Delta-predominant period. 3581 individuals (aged 18 to 100 years) from each timeframe were assessed. The seven most frequent symptoms were common to both variants. Within the first 28 days of illness, some symptoms were more common with Delta versus Alpha infection (including fever, sore throat, and headache) and some vice versa (dyspnoea). Symptom burden in the first week was higher with Delta versus Alpha infection; however, the odds of any given symptom lasting ≥ 7 days was either lower or unchanged. Illness duration ≥ 28 days was lower with Delta versus Alpha infection, though unchanged in unvaccinated individuals. Hospitalisation for COVID-19 was unchanged. The Delta variant appeared more (1.49) transmissible than Alpha. Re-infections were low in all UK regions. Vaccination markedly reduced the risk of Delta infection (by 69-84%). We conclude that COVID-19 from Delta or Alpha infections is similar. The Delta variant is more transmissible than Alpha; however, current vaccines showed good efficacy against disease. This research framework can be useful for future comparisons with new emerging variants.

## Introduction

Viruses mutate over time^1^, affecting transmissibility^2^, disease presentation^3^, and natural or vaccine-induced protective immunity.^4^ The Delta variant of SARS-CoV-2 (B.1.617.2) was identified in India in late 2020 and declared a ‘Variant of Concern’ in May 2021 by the UK^5^, the World Health Organization^6^, and the European Centre for Disease Control^7^, mainly due to evidence of increased transmissibility,^8,9^ possibly larger risk of hospitalisation,^10,11^ and conceivably less effectiveness of vaccination compared with previous variants^4,12,13^.

In the UK, the Delta variant rapidly became the dominant circulating form of SARS-CoV-2, (from 0.09% at the beginning of April 2021 to > 98% at the end of June 2021) displacing the Alpha (B.1.1.7) variant which concomitantly decreased from 98% to 1.67% (Supplementary Table S1, Supplementary Figure S1)^14^. On November 27, 2021, a new variant (Omicron B.1.1.529) emerged and spread rapidly, and became the predominant SARS-CoV-2 variant in UK after December 20, 2021. However, on 15 January 2022, Delta variant was still accounting for up to 40% new cases of SARS-CoV-2 infection in many European countries, and 20% cases in UK (https://www.gisaid.org/hcov19-variants/). Many other factors also changed contemporaneously, including SARS-CoV-2 prevalence, relaxation of lockdown restrictions, test access criteria, and the delivery of a mass vaccination campaign stratified by age, clinical vulnerability, and healthcare worker status.

We previously described COVID-19 profile, transmissibility, re-infection risk^15^, and vaccine effectiveness^16^ when the Alpha (B.1.1.7) variant was predominant. Here, we present these data after the Delta variant became predominant, and compare illness from Delta versus Alpha infection, in a large UK community cohort.

## Results

### Cohort description

44,718 adults testing positive for SARS-CoV-2 between December 28, 2020 and July 1, 2021, 22,699 had symptoms within requisite timeframes and logged sufficiently regularly for calculation of illness duration: 19,118 individuals when Alpha was predominant and 3581 when Delta was predominant. Demographic characteristics (after age, gender, and 1:1 matching) are presented in Table 1.

**Table 1:** Characteristics of UK individuals presenting with COVID-19, during periods of Alpha and Delta SARS-CoV-2 variant predominance.

	Cohort 1 (Alpha)	Cohort 2 (Delta)
Number of individuals	3581	3581
Age in years (median, (IQR))	40 (26–52)	40 (26–52)
Males (%)	41.0	41.0
BMI (kg/m2) (median, (IQR))	25.1 (22.4–29.0)	24.3 (21.9–27.8)
Vaccination status at time of positive test (numbers with 0/1/2 doses, (%))	3552 (99.2)/29 (0.8)/0 (0.0)	1183 (33.0)/881 (24.6)/1517 (42.4)
Diabetes (n, (%))	77 (2.2)	43 (1.2)
Asthma (n, (%))	488 (13.6)	465 (13.0)
Lung Disease (n, (%))	340 (9.5)	311 (8.7)
Kidney disease (n, (%))	17 (0.5)	20 (0.6)
Heart disease (n, (%))	45 (1.3)	48 (1.3)
Number of individuals with illness duration ≥ 28 days (n, (%))	380 (10.6)	311 (8.7)
Number of symptoms in the first week (median, (IQR))^a^	4 (3–6)	5 (3–7)

^a^Number of symptoms here considered from the 14 symptoms used in the analysis presented in Sudre et al. (viz.: fatigue, headache, dyspnoea, anosmia/dysosmia, persistent cough, sore throat, fever, myalgias, anorexia (‘skipped meals’), chest pain [otherwise undefined], diarrhoea, hoarse voice, abdominal pain and delirium)^17^.

### Illness profile

**Figure 1:**
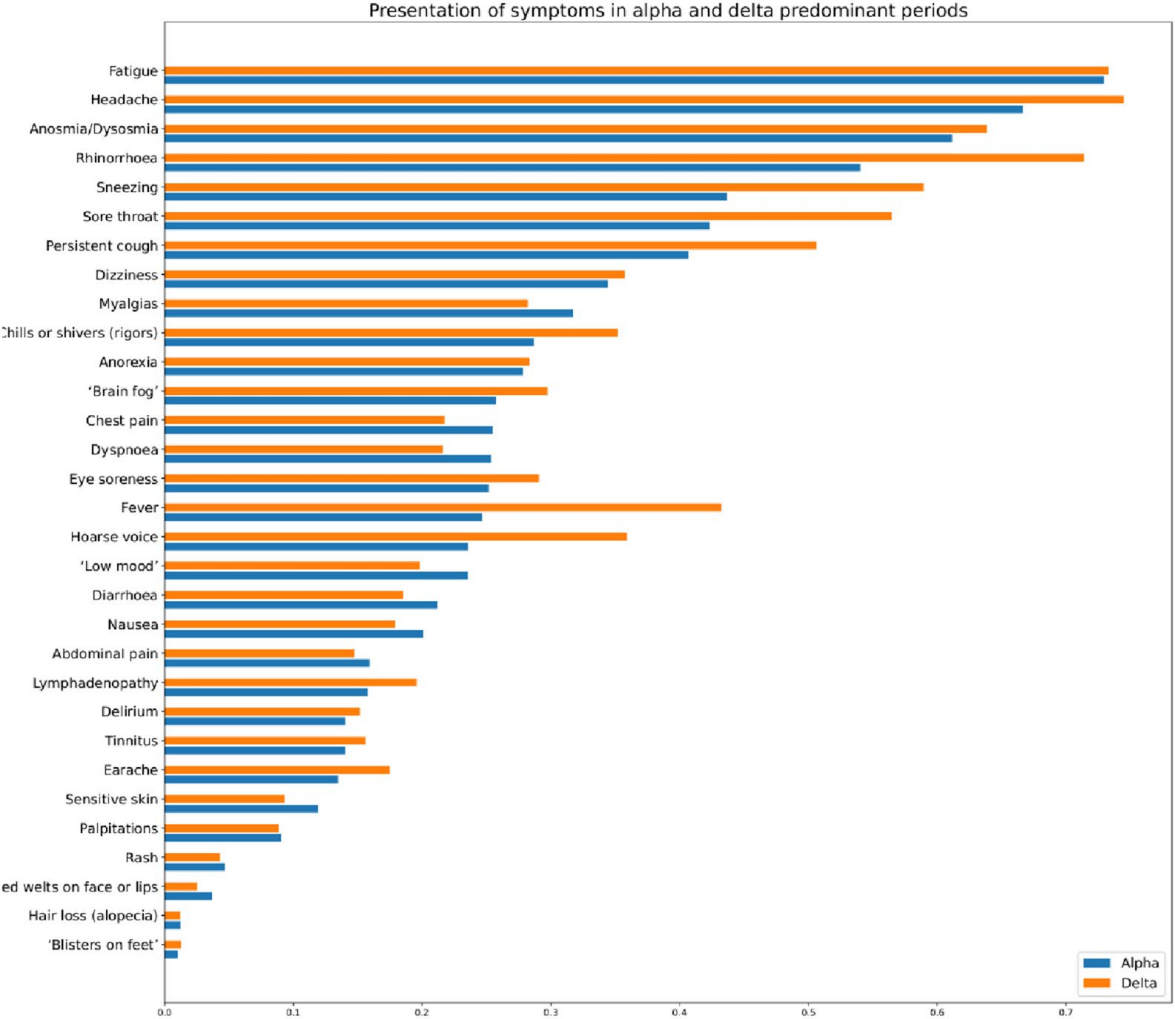
Prevalence of symptoms reported over the course of illness (up to 28 days) in individuals with COVID-19 during periods of SARS-CoV-2 Alpha or Delta variant predominance. Data are matched for age and sex but unmatched for local viral prevalence, lock-down restrictions, or vaccination.

Symptoms within the first 28 days of illness are presented in Fig. 1 and Supplementary Table S3 (descriptive data). All symptoms evidenced until day 28 had presented by day 21, in all individuals with both variants. The seven most reported symptoms were the same for Delta as for Alpha infection, though varied in prevalence: headache (75% vs. 67%), fatigue (73% in both), rhinorrhea (71% vs. 54%), anosmia/dysosmia (64% vs. 54%), sneezing (59% vs. 44%), sore throat (56% vs. 42%) and persistent cough (51% vs. 41%).

**Figure 2:**
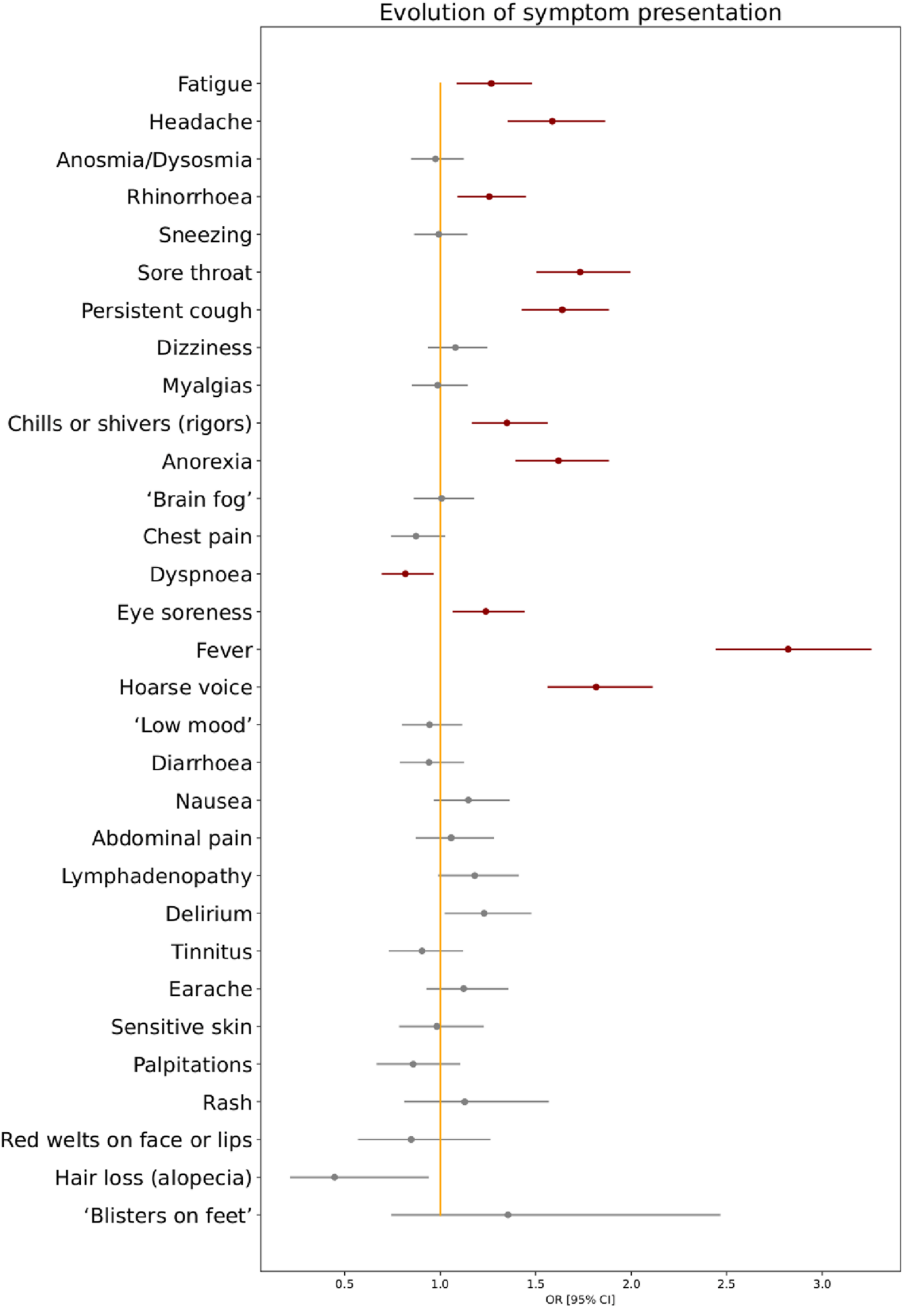
Odds ratios for any symptom presenting within the first 28 days of illness in individuals with COVID-19 during periods of SARS-CoV-2 Delta versus Alpha variant predominance. Age, gender, and vaccination.status are included as covariates in this analysis. Red markers encode statistical significance with α-value < 0.05, whereas grey markers encode non-significant differences, after correction for false discovery rate.

**Figure 3:**
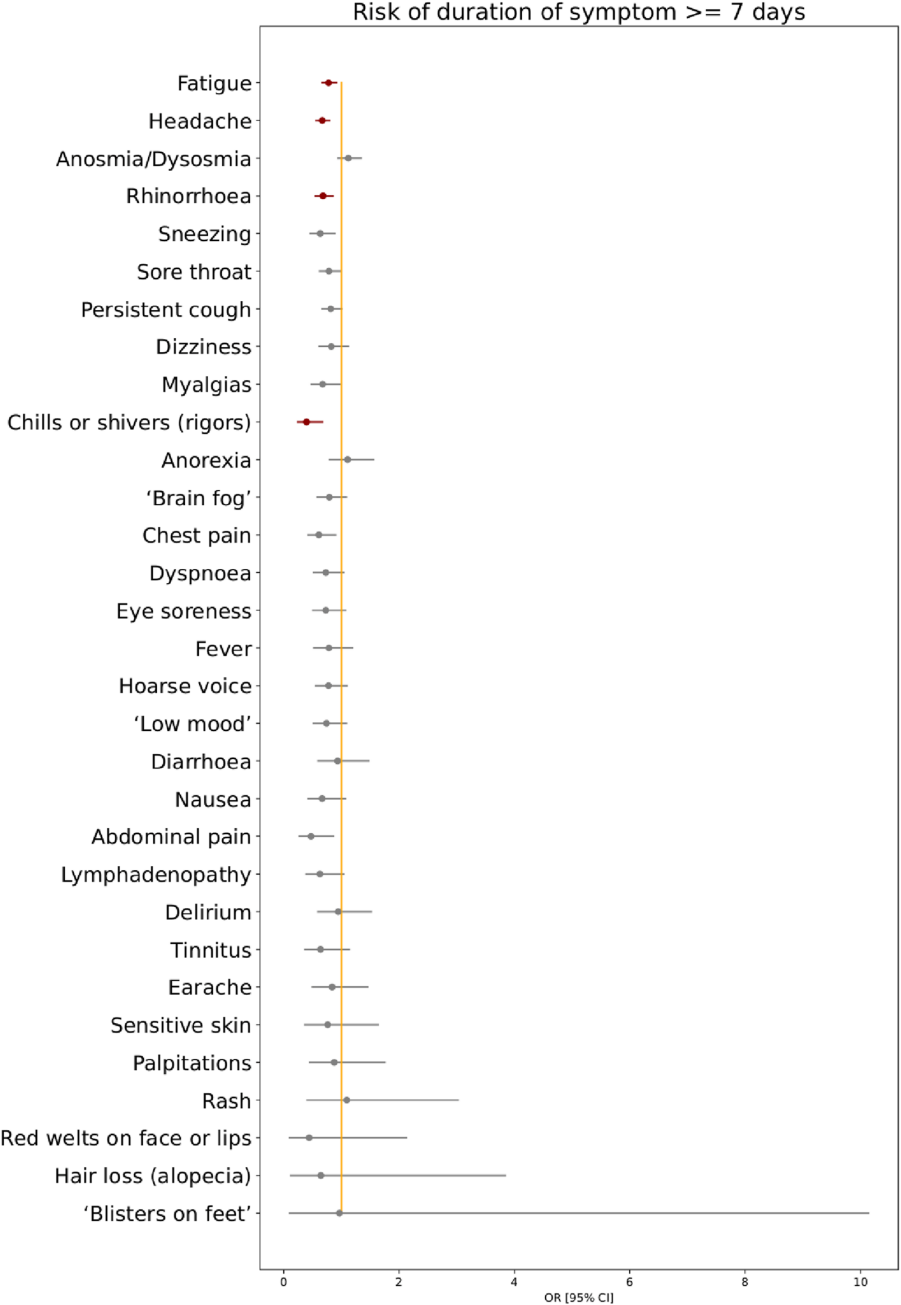
Odds ratios for risk of symptom duration ≥ 7 days for individuals with COVID-19 during periods of SARS-CoV-2 Delta versus Alpha variant predominance. Age, gender, and vaccination.status are included as covariates in this analysis. Red markers encode statistical significance with α-value < 0.05 (after FDR correction), whereas grey markers encode non-significant differences.

Correcting for age, sex, and vaccination status at time of positive test, and for false discovery rate, several symptoms were more common during the first 28 days of illness with Delta versus Alpha infection, including fever (OR 2.82 [95% CI 2.44–3.26]), hoarse voice (OR 1.82 [95% CI 1.56; 2.11]), sore throat (OR 1.73, [95% CI 1.5; 2]), and persistent cough (OR 1.64 [95% CI 1.43; 1.88]); conversely, the risk of shortness of breath was lower (OR 0.82 [95% CI 0.69; 0.96]) (Fig. 2, Supplementary Table S4). The odds of five or more symptoms in the first week of illness were higher with Delta versus Alpha infection, whether for the 14 symptoms analysed in Sudre et al.^17^ (OR 1.70 [95% CI 1.47; 1.95], *p* < 0.00005) or all symptoms (Supplementary Table S2) (OR 1.78 [95% CI 1.50; 2.11], *p* < 0.00005). However, the risk for any given symptom to last ≥ 7 days was either lower (chills, headache, rhinorrhea, fatigue) or unchanged (Fig. 3; Supplementary Table S4).

### Long illness duration

The risk of LC28 was moderately lower with Delta versus Alpha infection (8.7% [311/5581] vs. 10.6% [380/5581] of individuals, OR 0.69 [0.50; 0.94], *p* = 0.018 (considering the cohort overall, with age, sex and vaccination status included as covariates). In a sensitivity analysis considering only unvaccinated individuals (Table 1), there was a trend towards lowered risk of LC28 with Delta versus Alpha infection (OR 0.75 [0.54; 1.04], *p* = 0.087).

### Hospital presentation

Hospital care for COVID-19 was needed for 120 (3.4%) of 3581 individuals during the Delta period and 207 (5.8%) of 3581 during the Alpha period. Noting here that the UK vaccination campaign was stratified by age, clinical vulnerability, and health-care worker status, the risk of hospital presentation was moderately but not significantly lower for Delta versus Alpha infection, considered overall (OR 0.76 [95% CI 0.53; 1.11] *p* = 0.156, with age, sex and vaccination status included as covariates) or for unvaccinated individuals alone considered as a sensitivity analysis (OR 0.82 [95% CI 0.56; 1.20], *p* = 0.299).

### Transmissibility

**Figure 4:**
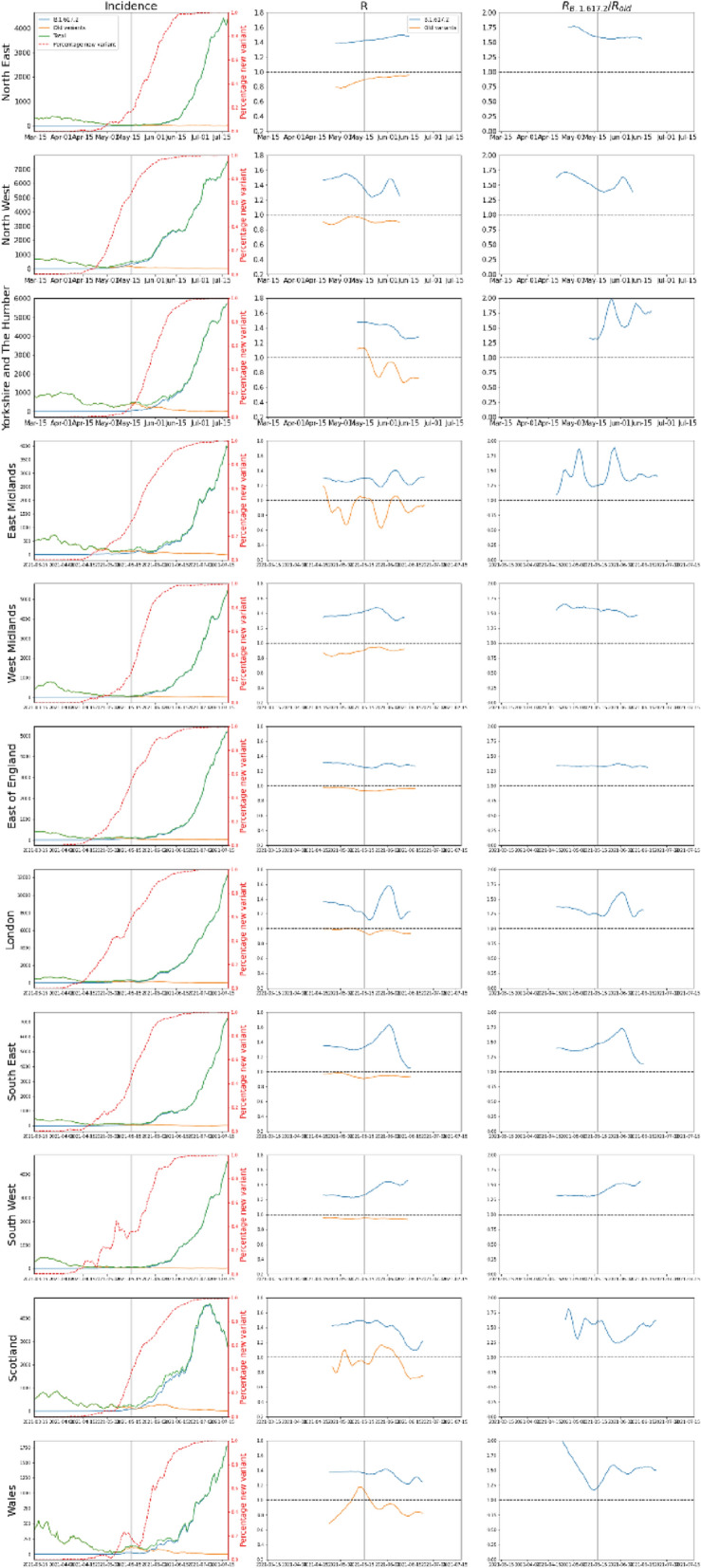
Incidence and R(t) for Delta and non-Delta variants. Left column shows total incidence, and incidence for each variant. Middle column shows R(t) for each variant. Rightmost column shows the ratio R_Delta/R_non-Delta, noting that in the timeframe considered non-Delta was predominantly Alpha. Vertical line indicates lifting of some lockdown restrictions on May 17, 2021.

Consistent with other studies, the Delta variant was more transmissible than Alpha, by 1.47 (95%CI 1.45–1.49) according to a population-weighted average (Table 2), noting wide confidence intervals and variation over time. Estimates per region agreed broadly with some regional variation (Fig. 4, Table 2).

**Table 2:** Increase in R(t) for Delta versus Alpha SARS-CoV-2 variants. R(t) is reported for regions in Great Britain (data from Northern Ireland insufficient to reliably conduct the analysis).

United Kingdom Region	Multiplicative R increase [mean (95% CI)]
North East	1.57 (1.55–1.59)
North West	1.47 (1.31–1.64)
Yorkshire and The Humber	1.69 (1.35–2.03)
East Midlands	1.44 (1.13–1.76)
West Midlands	1.52 (1.43–1.61)
East of England	1.34 (1.30–1.37)
London	1.36 (1.09–1.64)
South East	1.47 (1.11–1.84)
South West	1.47 (1.35–1.59)
Scotland	1.44 (1.18–1.69)
Wales	1.49 (1.31–1.66)

### Effect of the Delta variant on re-infection

**Figure 5:**
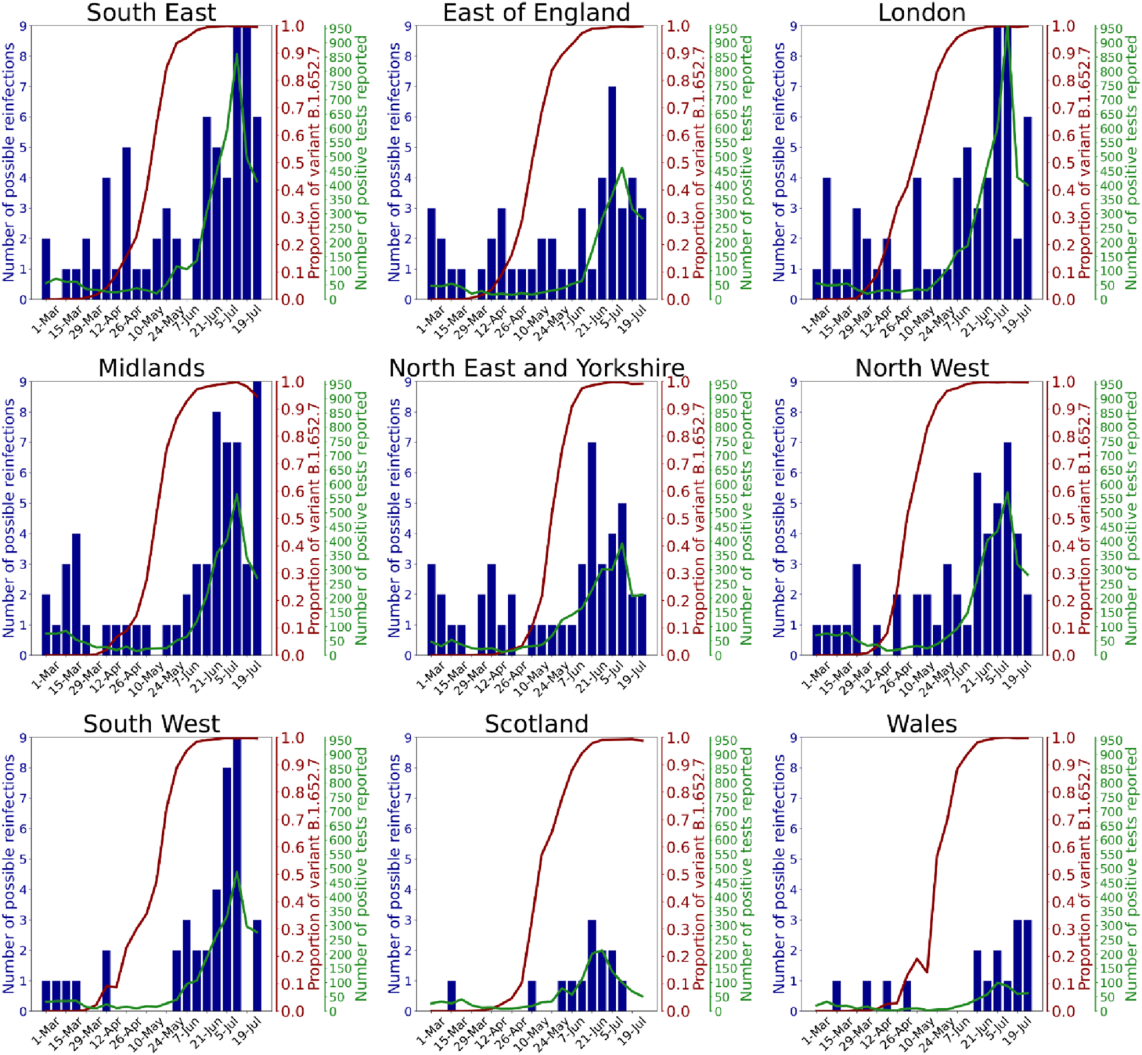
Regional graphs presenting evolution of numbers of reported natural reinfection with SARS-CoV-2. Reported natural reinfection is charted over time in weeks (starting from March 1, 2021) (x-axis). The blue bars graph the absolute number of cases with re-infection. The red line graphs the proportion of the Delta variant among circulating SARS-CoV-2 (COG UK—Supplementary Table S1). The green line graphs the total numbers of positive tests reported by ZOE COVID Symptom Study app users. Data are combined for East and West Midlands, and for Yorkshire and the North East.

Figure 5 shows the (small) absolute numbers of re-infections across regions, with: a) the number of positive tests reported by app users; and b) the Delta variant as a proportion of circulating SARS-CoV-2, over time. Spearman correlations between reinfection and positive test incidence ranged from 0.46 in the South East to 0.83 in the Midlands. Correlations between reinfection and Delta variant proportions in each region were lower, ranging from 0.41 in the North East and Yorkshire to 0.69 in the North West. In most regions, the correlation of reinfections with the number of reported tests was higher than the correlation of reinfections with the proportion of Delta variant. Supplementary Table S5 presents characteristics of the bootstrapped distribution (100 samples) of correlations for each region over time. Thus, the rise of SARS-CoV-2 infection during the time of Delta predominance correlates more closely with the rise of incidence of new cases per se, rather than the rise in proportion of cases due to the Delta variant specifically.

### Post-vaccination infection during the Delta period

402,191 app users aged 20–65 years were vaccinated with BNT162b2 (1st dose only: 33,171, 2nd dose: 117,091) or ChAdOx1 nCoV-19 (1st dose only: 59,663, 2nd dose: 192,266) and reported at least one PCR or LFAT test after vaccination between May 26 and July 1, 2021. A positive result was reported by 1723 of 92,834 (1.86%) who had received one dose, and 1722 of 309,357 (0.56%) who had received two doses. Data were compared to 25,395 unvaccinated time-matched participants, in whom 1361 (5.36%) individuals reported a positive test.

**Figure 6:**
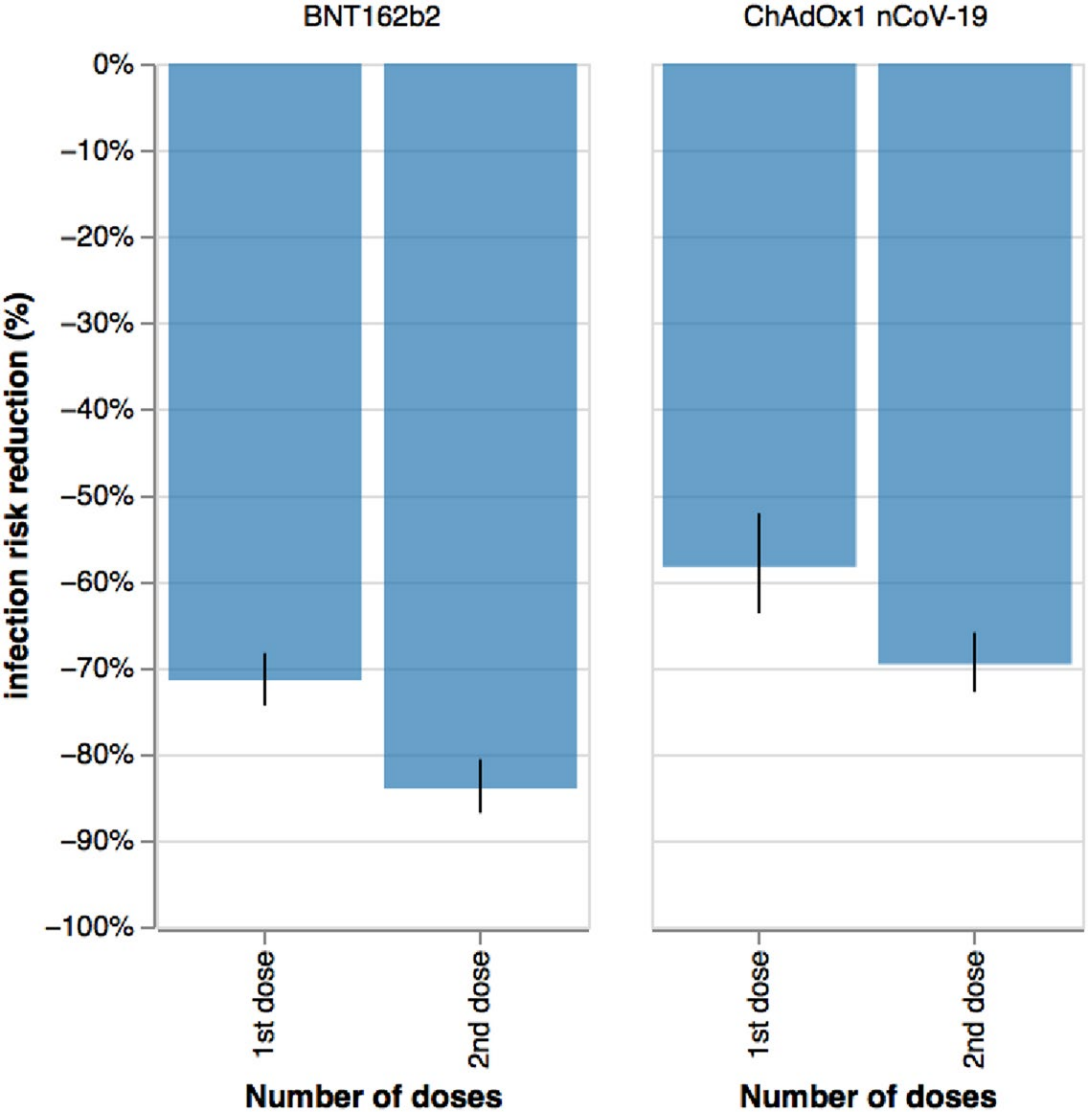
Infection risk reduction (in %) with Delta variant 14–60 days after vaccination after one or two doses of either BNT162b2 or ChAdOx1 nCoV-19 vaccines.

After adjustment for population differences in the vaccinated groups using Poisson regressions as described in Methods, the risk reduction of post-vaccination infection after first dose (considered 14–60 days after first dose) was − 71.5% (95% CI − 74.4 to − 68.3) with BNT162b2 and − 58.3% (95% CI − 63.7 to − 52.1) with ChAdOx1 nCoV-19, compared with unvaccinated individuals. The risk reduction was even larger in fully vaccinated individuals (considered 14–60 days after second dose): − 84.1% [95% CI − 86.9 to − 80.6] with BNT162b2, and -69.6% [95% CI − 72.9 to − 65.9] with ChAdOx1 nCoV-19, compared with unvaccinated controls (Fig. 6).

## Discussion

Our large-scale community-based UK study has shown that COVID-19 is clinically similar whether due to Alpha or Delta variants. Ten of 31 symptoms were more common with Delta infection and one with Alpha infection. Although the burden of symptoms in the first week was higher with Delta infection, duration of many individual symptoms was shorter; fewer individuals experienced illness lasting more than 28 days—though saliently this was unchanged in unvaccinated individuals; and there was a trend towards fewer hospital presentations. These observations need to be interpreted in the context of increasing vaccination of the UK population, along with many other environmental and societal changes.

Few studies of COVID-19 due to the Delta variant are available for comparison. One study of 27 infected young individuals reported symptoms in 22 (81%), with the commonest symptoms fever (41%), cough (33%), headache (26%), and sore throat (26%) (duration of illness not reported)^18.^ Other studies report smaller cohorts. The REACT-1 study Round 14 report (UK data during September 2021, with Delta variant the predominant UK variant) showed a weighted prevalence of individuals testing positive varied greatly by age (0.29% in adults aged > 75 years compared to 2.55% in teenagers and 2.32% in children aged 5–12 years), noting high vaccination rates in older individuals and little or no vaccination in younger age groups at the time of this report^19^. However, data on symptoms (duration and/or prevalence) were not reported.

The risk of LC28 was lower with Delta (8.7%) versus Alpha infection (10.6%), although not statistically different in unvaccinated individuals. These results are similar to our previous paper using similar methodology, for individuals infected during the first UK pandemic wave (13.3%)^17^. The Post COVID syndrome (Long COVID) is defined as illness duration > 12 weeks after likely SARS-CoV-2 infection (https://www.nice.org.uk/guidance/NG188). Our census dates preclude our ability to compare illness duration beyond 28 days between our two cohorts. Estimates of prevalence of the Post-COVID syndrome are difficult, as many studies lack appropriate control groups. A recent meta-analysis of UK longitudinal cohort studies suggested the post-COVID syndrome was present in 1.2–4.8% of individuals^20^, similar to recently published figures from the Office for National Statistics (ONS) (1.9% of the UK population self-reporting long COVID (diagnosis otherwise unverified) as of October 2, 2021, although only 71% had (or suspected they had) COVID-19 12 weeks earlier (https://www.ons.gov.uk/peoplepopulationandcommunity/healthandsocialcare/conditionsanddiseases/bulletins/prevalenceofongoingsymptomsfollowingcoronaviruscovid19infectionintheuk/4november2021).

We showed a marked increase in transmissibility with the Delta versus non-Delta (i.e., Alpha) variant, noting wide confidence intervals. This analysis does not take into account prior natural infection or vaccination rates within the community; and is likely a combination of both the Delta variant’s transmission advantage and its potential ability to evade immunity (whether induced by vaccination or prior natural infection with Alpha or other strains). This estimated increase in transmissibility is greater than we previously estimated for Alpha versus earlier variants using the same methodology [1.35 (95% CI 1.02–1.69)]^15^ noting again pertinent differences (e.g., viral prevalence, lockdown restrictions) between the current and previous studies. Estimates in both studies assume that incidence estimated from app users can be made representative of the wider population, using stratification by age and vaccination status. However, other factors such as behavior and socio-economic status are not corrected for by this analysis. Other studies have also identified higher Delta transmissibility, resulting in rising incidence particularly in young unvaccinated age groups, higher re-infection rates, and a higher viral load in infected individuals^21^. Here we note the REACT-1 study report of an exponential increase in infections in children aged 5–17 years in September 2021, coinciding with return-to-school, with most school-age children unvaccinated at this time.

Our study found that for most regions of the UK, the correlation of reinfections with the number of reported positive tests (i.e., incidence of cases) was higher than the correlation of reinfections with the proportion of Delta among the circulating variants (i.e., incidence of variant). In other words, the rise in COVID-19 correlated more closely with increase in prevalence of SARS-CoV-2 overall rather than the increased proportion of circulating SARS-CoV-2 due to the Delta variant. SARS-CoV-2 infection provides substantial and persistent immunologic protection for at least several months for most individuals, with a recent systematic review suggesting a risk reduction of reinfection of > 90%, similar to vaccination, and evident for at least 10 months^22^. However, this may not be uniform across the population. A study of tested individuals followed prospectively for at least 3 months demonstrated a protective effect after prior infection of 80.3% for younger individuals (aged 20–59 years) but only 67.4% for older individuals (aged ≥ 60 years), with lower levels of protection in individuals associated with a long-term care facility and/or who had milder initial disease^23^. A study of Danish healthcare workers^24^ found small absolute numbers of re-infected individuals (5 of 750 seropositive individuals over 5–6 months). However, 5% of previously seropositive individuals reverted to seronegative status, associated with older age and fewer symptoms with initial infection (noting here that the relationship between antibody titre and subsequent infection risk is currently unclear). In July 2021 UK governmental figures estimated an adjusted odds ratio of reinfection risk from the Delta variant versus the Alpha variant as 1.5. However, this varied according to time since initial infection: the odds ratio was not elevated if initial infection was < 180 days earlier (adjusted odds ratio = 0.8), but was higher if initial infection was ≥ 180 days earlier (adjusted odds ratio = 2.4) (https://assets.publishing.service.gov.uk/government/uploads/system/uploads/attachment_data/file/1005517/Technical_Briefing_19.pdf).

Our observational data support effectiveness of both BNT162b2 and ChAdOx1 nCoV-19 vaccines against the Delta variant. Both reduced the risk of testing positive during the Delta period, evident after the first and enhanced after the second dose^25^. These figures are similar to our previous results when the Alpha variant was predominant^15^. We have an inherent bias due to the nature of the UK vaccine rollout, whereby health-care workers, elderly people, and clinically vulnerable individuals were prioritised before the younger population, creating unbalanced demographic characteristics between vaccinated and unvaccinated populations. Moreover, we cannot compare vaccination effectiveness against Alpha versus Delta variants, given the many differences between the two timeframes. Although we attempted to adjust for some of these differences using Poisson regression, behavioural factors are difficult to capture. For example, individuals vaccinated earlier may have changed their behaviours over concern of possible waning antibody status and possible reducing immunity^26^. However, our results concord with vaccination trial data^27,28^, and provide support for ongoing vaccination campaigns internationally. Previous data have shown that vaccination is associated with significant reduction in risk of hospitalisation and disease progression to death or mechanical ventilation in individuals with COVID-19^12,29^. Our data similarly showed a trend towards fewer hospital presentations. Later analyses during further waves of the pandemic will be useful here with the methods and approaches described here similarly applied to assess the impact of emerging variants.

We acknowledge the limitations of our observational study. Self-reported data from a mobile phone app may disproportionately represent more affluent populations and can introduce information bias and/or effect bias, although previous work from the CSS has shown that our self-reported data aligns well with surveys designed to be representative of the population^30^ and smartphone ownership in the UK is extensive with little evidence that this varies greatly across socio-economic groups. Further, 1600 of 7162 individuals were proxy-reported, which may also affect symptom reporting, although the proportion of proxy-reported individuals as a percentage of total individuals was the same for both time-periods (22.4%). Participants could only report a positive test and we cannot confirm the actual variant causing infection, although our assumptions of Delta and Alpha infection are strongly supported by UK-COG surveillance variant testing. During the study, both overall numbers and individual app users fluctuated in their participation in the CSS-app, potentially for many factors including mass-media information, summer vacation, and perception of relevance. Our populations were matched for age and sex but not BMI; and we note higher diabetes prevalence and BMI in the Alpha cohort. Relevantly, vaccination was not only tiered by age but also to those with co-morbidities including diabetes. As mentioned, the timeframes of Alpha and Delta variant predominance differed with respect to guidance on social distancing and behavior in public spaces, highly likely to affect viral diffusion in the population, thus affecting our transmission calculations. Last, vaccine effectiveness could only be determined in tested individuals, noting that we do not have information regarding the reason for testing in these individuals. We were also only able to assess individuals in the age range of 20–65, in order to avoid unbalanced case/control data. Relevantly, early post-vaccination symptoms can mimic COVID-19^31^ but may not necessarily trigger testing. Here, our previous work showed that vaccinated individuals are more likely to have post-vaccination systemic symptoms after a previously positive test compared to those without known past infection (odds ratios 2.3–4.0), which may bias presentation for SARS-CoV-2 testing post-vaccination.

## Conclusions

The clinical presentation of COVID-19 due to the Delta variant is similar to illness caused by the Alpha variant: although symptom burden in the first week is modestly higher, individual symptom duration was either the same or shorter, and the risk of LC28 was lower. The Delta variant was more transmissible than the preceding predominant variant (i.e., Alpha) but did not increase the risk of reinfection per se. The risk of infection in fully vaccinated individuals was reduced by both BNT162b2 and ChAdOx1 nCoV-19, compared with unvaccinated controls, confirming good vaccine efficacy against the Delta variant and supporting the ongoing anti-SARS-CoV-2 vaccination campaign internationally.

## Methods

### COVID Symptom study

Prospective longitudinal observational data were collected as part of the King’s College London [KCL]/ZOE COVID Symptom Study, using the ZOE COVID Study App^32^ (Supplementary Information). Briefly, upon enrolment users provide baseline demographic and health information, and subsequently are prompted daily to record symptoms (or their absence) through direct questioning (Supplementary Table S2) and free text, any SARS-CoV-2 testing and corresponding result, vaccination details, and any hospital presentation. Users can also proxy-report for others. The current study was drawn from approximately 1 million UK app users who logged data at least once between December 28, 2020 to July 1, 2021. Data were extracted and curated through ExeTera software^33^.

Ethics approval was granted by the KCL Ethics Committee (LRS-19/20-18210). To ensure informed consent, at registration with the app, all participants were shown informative documentation, and were offered to provide consent for their data to be used for COVID-19 research. Governance was specifically granted for use of proxy-reported data. Research was performed in accordance with the relevant guidelines and regulations, and particularly in full compliance with the Declaration of Helsinki and further updates.

Data from all UK adult participants aged 18 to 100 years (including proxy-reported individuals) who logged a positive PCR or lateral flow antigen test (LFAT) for SARS-CoV-2 between December 28, 2020 to July 1, 2021 were considered. As previously^17^, individuals were considered to have COVID-19 if SARS-CoV-2-associated symptoms were reported (or proxy-reported) (Supplementary Table S2) between two weeks before and one week after positive testing. Data were included for individuals who reported at least weekly, from first symptom report until returning to symptom-freedom or until reporting ceased^22^.

Data were compared between two time periods: December 28, 2020 to May 6, 2021, when the Alpha variant was the predominant circulating SARS-CoV-2 strain (proportion of sequenced strains: > 75% from December 28, 2020, reaching > 95% by February 3 2021, and remaining > 75% until 28 April); and May 26, 2021 to July 1, 2021, when the Delta variant was the predominant strain (> 75% from May 26, reaching > 95% by June 9 and > 99% from June 30 to data census date) (Supplementary Table S1). Individuals logging a positive test did not have variant confirmation by sequencing; thus, illness within these two timeframes was attributed to the predominant circulating variant. Terminology herein reflects this assumption.

Through an Euclidean distance-based algorithm^34^, individuals with Delta infection were matched 1:1, based on their age and sex, with individuals with Alpha infection. We were unable to match for SARS-CoV-2 prevalence, tiered lockdown restrictions, or vaccination rates, which varied widely across the community and with time during this study.

Symptom data were censored at August 5, 2021, 35 days after last inclusion date for testing positive with Delta infection, allowing at least 28 days’ symptom evaluation for all individuals. Symptoms were considered over the entire illness, which by virtue of illness definition could extend outside SARS-CoV-2 testing date boundaries (a maximum of two weeks before and five weeks after testing, allowing for individuals whose illness started up to a week after positive test). To allow for symptom waxing and waning, individuals who returned a healthy report but subsequently logged as symptomatic within seven days of their last unhealthy report were considered unwell from their initial illness, with per-symptom and illness duration calculated accordingly.

We ascertained odds of a given symptom developing within 28 days of illness; and odds of each symptom lasting ≥ 7 days, corrected for age, sex and vaccination status (unvaccinated, 1 dose, 2 doses), with a given vaccination considered valid after 14 days (allowing for evolving immunity). We used false discovery rate to account for multiple comparisons. We assessed risk of illness duration ≥ 28 days (LC28) and hospital presentation (admission or emergency room attendance), in the cohort overall (similarly adjusted for age, sex and vaccination status) and in unvaccinated individuals alone, as a sensitivity analysis.

### Transmissibility

We used data from COVID-19 Genomics UK Consortium (COG-UK) to extract time-series of the percentage of daily positive SARS-CoV-2 testing from the Delta lineage in Scotland, Wales, and each of nine National Health Service (NHS) regions in England. Northern Ireland was excluded due to low sample numbers in the COG-UK dataset. The COG-UK data are produced by sequencing a random sample of positive PCR tests from the general community.

Daily SARS-CoV-2 incidence data for Scotland, Wales, and each NHS region in England were estimated from March 14 to August 8, 2021, using CSS app data and previously described methodology^35^. The method uses both positive SARS-CoV-2 test results and symptom reports from app users, to estimate incidence. Data are stratified by age and vaccination status to ensure estimates made from the CSS app population are representative of the wider population.

Using COG-UK data to estimate the proportion of Delta in circulation in each region per day, incidence estimates were decomposed into two incidence time-series per region, one for ‘non-Delta’ (in the timeframe considered here, predominantly Alpha [Supplementary Table S1]) and one for Delta, assuming that the two incidence time-series should sum to match total incidence. R(t) was estimated separately for non-Delta and Delta variants, using previously described methodology^35^. Briefly, we used the relationship I_t+1_ = I_t_ exp(μ (R(t) – 1)), where 1/μ is the serial interval and I_t_ the incidence on day t. We modelled the system as a Poisson process and assumed the serial interval was drawn from a gamma distribution with α = 6.0 and β = 1.5; and used Markov Chain Monte-Carlo to estimate R(t). We compared both multiplicative and additive differences of the new and old R(t) values for days when the Delta proportion in a region was > 3%.

### Reinfection during rise of Delta variant

Reinfection was defined as previously^36^ (presence of two positive PCR or LFAT tests separated by > 90 days, with an asymptomatic period of ≥ 7 days before the second positive test). To assess risk of reinfection during the Delta variant timeframe we performed ecological studies for each region, examining the Spearman correlation between the proportion of circulating SARS-CoV-2 due to Delta (Supplementary Table S5, Supplementary Figure S1) and number of reinfections per week over time, assessed from 10 weeks prior to Delta prevalence of 25% until 10 weeks after Delta prevalence of 75% (22 weeks); and between the number of positive tests reported through the app and the number of reinfections. We compared the bootstrapped distributions of these two correlations in each region, using the Mann–Whitney U test (Supplementary Table S5).

### Post-vaccination infection during Delta period

We analysed 515,138 app users who reported vaccination with BNT162b2 (BioNTech-Pfizer) or ChAdOx1 nCoV-19 (Oxford-Astra Zeneca) and were subsequently tested for SARS-CoV-2 (PCR or LFAT) 14–60 days after either first or second vaccination (assessed separately) after 26 May 2021^36^. Age was restricted to 20–65 years, as most individuals > 65 years were vaccinated and most individuals < 20 years unvaccinated during the time of analysis, biasing the control groups for these ages. Users who had reported SARS-CoV-2 infection previously were excluded. Unvaccinated users reporting SARS-CoV-2 test results in the same or following week as a vaccinated app user served as controls. In the event of multiple tests logged for an individual vaccinated user, either the first positive or the last negative result was selected. For each vaccine and per dose, we modelled rates of positive testing in vaccinated versus unvaccinated individuals, using Poisson regressions adjusting for number of tests, age, co-morbidities, sex, healthcare worker status, obesity, and weekly incidence in the community (by controlling for the date of the test). The adjusted risk reduction was then calculated as *RR* = *riskratio*_*i,n*_ − *1*, where *i* is the vaccine type, and *riskratio* is the ratio of infection rates in vaccinated individuals compared to unvaccinated individuals, derived from our Poisson model.

### Ethics

Ethics approval was granted by KCL Ethics Committee (reference LRS-19/20-18210).

## Supplementary Information

Supplementary Information.

## Data Availability

Data collected in the COVID Symptom Study smartphone app can be shared with other health researchers through the UK National Health Service-funded Health Data Research UK and Secure Anonymised Information Linkage consortium, housed in the UK Secure Research Platform (Swansea, UK). Anonymised data are available to be shared with researchers according to their protocols in the public interest, and an administrative fee might apply. Link at https://web.www.healthdatagateway.org/dataset/594cfe55-96e3-45ff-874c-2c0006eeb881. Information on anonymized SARS-CoV-2 test results is recorded as positive/negative/invalid, and sequencing data were not captured.

## Abbreviations

CI: Confidence interval
COVID-19: Coronavirus disease 2019
IQR: Inter-quartile range
LC28: Long COVID-19 with duration of symptoms ≥ 28 days
LFAT: Lateral flow antigen test
ONS: Office for National Statistics (UK)
OR: Odds Ratio
PCR: Polymerase chain reaction
SARS-CoV-2: Severe acute respiratory syndrome‐related coronavirus 2
UK: United Kingdom of Great Britain and Northern Ireland
WHO: World Health Organisation
